# Uptake rates and attitudes to influenza and COVID-19 vaccination in pregnancy – a prospective cohort study

**DOI:** 10.1007/s11845-023-03428-0

**Published:** 2023-06-20

**Authors:** Sarah M. Kelly, Orla Bracken, Tariq Bholah, David A. Crosby

**Affiliations:** 1 Department of Obstetrics and Gynaecology, National Maternity Hospital, Holles Street, Dublin 2 Dublin, Ireland; 2 Department of Obstetrics and Gynaecology, UCD School of Medicine, University College Dublin, Dublin, Ireland

**Keywords:** COVID-19, Influenza, Vaccination, Pregnancy, Vaccine-hesitancy

## Abstract

Influenza and COVID-19 are highly prevalent RNA viruses. Pregnancy increases the frequency of severe maternal morbidity and mortality associated with these viruses. Vaccination plays an important role in protecting pregnant women and their infants from adverse outcomes. In this prospective study, we aimed to determine the vaccination uptake rate for influenza and COVID-19 in a pregnant population and to explore reasons why women remained unvaccinated. A prospective cohort study was conducted over a two-week period in December 2022 in the National Maternity Hospital, Dublin. There were 588 women surveyed over the 2-week period. Overall, 377 (57%) were vaccinated that year for seasonal influenza, a significant rise from 39% in a similar study in 2016. The majority (n = 488, 83%) of women reported receiving at least one COVID-19 vaccine. However only 132 (22%) received a COVID-19 vaccine in pregnancy, despite 76% (n = 466) stating they would be happy to receive it. Factors such as age, obesity, co-morbidities, ethnic group, and type of antenatal care received were shown to influence vaccination rates. We recommend that the importance of vaccination be stressed regularly to eligible patients at their antenatal clinic visits and where possible combining influenza/COVID-19 vaccination on the same day to improve uptake.

## Introduction

Influenza and COVID-19 are highly infectious respiratory diseases caused by the RNA viruses Influenza A or B and SARS-CoV-2, respectively. Transmission is mediated by human-to-human contact via respiratory droplets. Influenza outbreaks are typically seasonal with epidemics occurring during the winter months [1]. Although COVID-19 is a relatively new disease with the pandemic being declared in March 2020, recent research has demonstrated that a seasonal-like pattern is emerging with the virus in a similar fashion to influenza [2]. Epidemiological evidence has shown that pregnancy is associated with increased susceptibility to viral infections and subsequent development of serious complications. This is likely due to the physiological changes within the respiratory, endocrine and immune systems during pregnancy and the puerperium [3].

Influenza and COVID-19 infection in pregnancy are independently associated with increased rates of adverse events such as hospitalization, intensive care admission, preterm births, stillbirth and maternal death [4]. The most recent data from the UKOSS study of COVID-19 in pregnancy has shown that 98% of patients admitted to hospital with symptomatic COVID-19 were unvaccinated. Furthermore, only 3 of the 235 patients admitted to ICU were vaccinated, however, none had received their second booster dose. The MBRACE-UK Confidential Enquiry report presents triennial findings of maternal mortality in the UK and Ireland. The most recent report showed that 7 maternal deaths have been attributable to influenza from 2014–2020 [5]. Provisional reporting from MBRACE-UK up until September 2021 has shown that a total of 33 pregnant woman have died from COVID-19 since the start of the pandemic with 85% of these being unvaccinated. Furthermore, maternal mortality associated with COVID-19 seems to be increasing with each new wave of the virus with the delta wave proving to have the highest mortality rate [6].

At present, the Royal College of Physicians of Ireland (RCPI), Irish Health Service Executive (HSE) and the Royal College of Obstetricians and Gynaecologists (RCOG) all recommend inactivated influenza and mRNA COVID-19 vaccination in pregnancy to reduce maternal and perinatal morbidity and mortality [7–9]. In Ireland, the National Immunisation Advisory Committee (NIAC) have updated their 2023 COVID-19 vaccination strategy recommendations. They advise that all pregnant women should receive a booster in pregnancy if it is more than six months since their previous COVID-19 vaccine or infection. Although COVID-19 vaccination is safe at any time in pregnancy, to obtain maximum benefit from the vaccine, it should ideally be given between 20–34 weeks’ gestation [10]. Multiple studies have shown that both the COVID-19 RNA vaccine and inactivated influenza vaccine are safe to give in pregnancy and are associated with a significant reduction in adverse maternal outcomes [11, 12]. Furthermore, both vaccines have been shown to offer protection in infants up to six months old and reduce viral infection in infancy through transfer of maternal antibodies [13]. Despite this, vaccination rates of influenza and COVID-19 remain low in expectant mothers while barriers to vaccination remain poorly understood [12]. A recent systematic review, which included over 700,000 pregnant women, found that COVID-19 vaccination uptake in pregnancy was as low as 27.5% [14]. A similar study performed at the National Maternity Hospital (NMH) in 2016 showed that influenza vaccination uptake in pregnancy was 39%, which was lower than the average UK and US vaccination rates. The study attributed a number of factors to the low vaccination rates such as poor education regarding influenza risk and patient concerns on vaccine safety in pregnancy [15].

In this study, we aim to evaluate influenza and COVID-19 vaccination uptake rates in pregnancy. Furthermore, we will assess attitudes towards vaccination and identify barriers to vaccination in expectant mothers.

## Methods

A prospective cohort study was conducted at the National Maternity Hospital, a tertiary maternity referral hospital, over a two-week period in December 2022. A voluntary survey was distributed to patient’s attending ‘normal’ risk antenatal clinics and ‘high’ risk maternal medicine and diabetic antenatal clinics. Patient demographics, comorbidities, influenza and COVID-19 vaccination status were recorded. In patients who answered ‘yes’ to receiving either vaccine, it was recorded whether this was pre-pregnancy/during pregnancy and the gestational age at the time of vaccination was noted. In those who had answered ‘no’ to receiving either vaccine, the reason why was recorded. Finally, attitudes towards COVID-19 vaccination in pregnancy were assessed by asking if expectant mothers would be happy to receive a COVID-19 vaccine during pregnancy and, if so, at which gestation. Data was compiled and analysed using Excel 2010.

## Results

There were 588 women studied over the two-week period. Most of the women were aged between 30 and 35 years, and between 35 and 40 years (35% and 32% respectively). There were 280 (48%) nulliparous women and 308 (52%) multiparous women. The majority were White (82%). The mean body mass index (BMI) was 27.6 kg/${\mathrm{m}}^{2}$ and the mean gestational age when studied was 28 weeks gestation. Five hundred and twenty-nine subjects (90%) attended for publicly-funded obstetric antenatal care, forty-eight (8%) attended for semi-private funded obstetric care and eleven (2%) attended for private-funded obstetric antenatal care. One hundred and eighty-five women (31%) reported having medical conditions such as thyroid issues (n = 53), diabetes (type1/2/gestational diabetes mellitus n = 62), asthma (n = 32), hypertension (n = 12), heart disease (n = 7), renal disease (n = 3) and other (n = 39).

Table 1 summarises the demographic data of Influenza Vaccinated and COVID-19 Vaccinated women (before and during pregnancy). Notably, the rates of influenza and COVID-19 vaccination among public antenatal care were 54% (n = 287/529) and 81% (n = 429/529) respectively compared to 85% (n = 50/59) and 100% (n = 59/59) respectively among semi-private/private antenatal care. Vaccination rates were higher in women with medical co-morbidities than those without co-morbidities. In women with diabetes (type 1/2/gestational), the influenza vaccine uptake was 79% (n = 49/62) and COVID-19 vaccine uptake was 89% (n = 55/62). In women with asthma, the influenza vaccine uptake was 63% (n = 20/32) and COVID-19 vaccine uptake was 84% (n = 27/32). COVID-19 vaccination uptake rates in pregnancy increased with increasing age, increasing BMI and the presence of co-morbidities. COVID-19 vaccination uptake rates in pregnancy were highest amongst women attending private antenatal care (45%) and amongst Chinese ethnicity (38%).

**Table 1:**
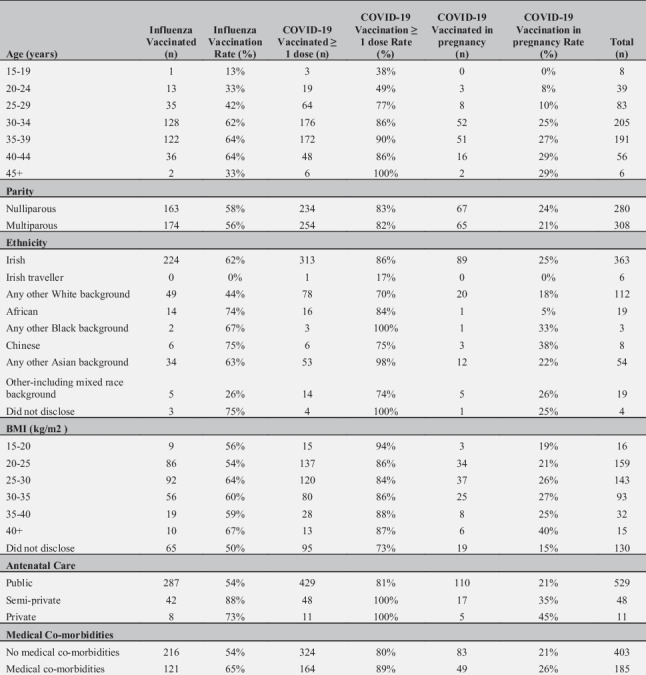
Demographic Table for Influenza Vaccinated (n = 337), COVID-19 Vaccinated (≥ 1 dose, n = 488) and COVID-19 Vaccinated in pregnancy (n = 132), out of 588 women surveyed

Overall, 337 (57%) received the influenza vaccine in 2022, with 3% receiving it before pregnancy and the remainder receiving it during pregnancy. The mean gestation to receive the vaccine was 24 weeks gestation. A further 10% (n = 58) reported that they planned to receive the vaccine during pregnancy. One hundred and ten (19%) women said they would prefer not to receive the influenza vaccine, 24 (4%) felt it was unsafe in pregnancy, 12 (2%) were not aware it was recommended and 6 (1%) refused it due to allergies.

**Fig. 1:**
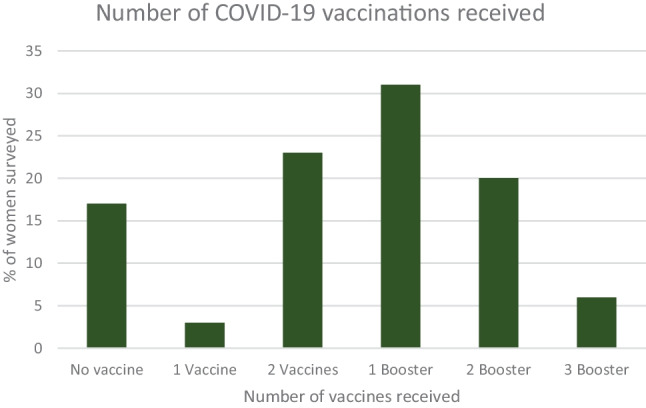
Graph representing the number of COVID-19 vaccinations received by the total cohort, n = 588

The majority of women reported a history of COVID-19 infection (n = 444, 76%) and 66 (22%) women reported infection in the last four months. Figure 1 shows the number of COVID-19 vaccines received by women, revealing that most women had received at least one COVID-19 vaccine (n = 488, 83%).

**Fig. 2:**
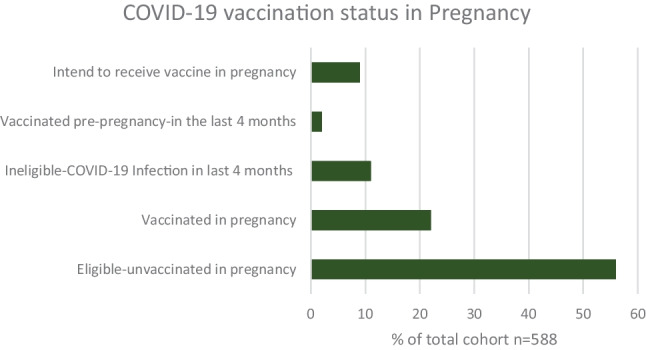
Graph demonstrating the COVID-19 vaccination status of the cohort surveyed, n = 588

The most common vaccine received was the Pfizer vaccine (n = 408, 84%), followed by the Moderna (n = 59, 12%) and the AstraZeneca vaccine (n = 51, 10%). The mean gestation for COVID-19 vaccine in pregnancy was 24 weeks. However, most women were vaccinated pre-pregnancy (n = 356/588, 61%) and only one-hundred and thirty-two women (n = 132/588, 22%) received a vaccine during pregnancy. Figure 2 depicts the COVID-19 vaccination status of the cohort. It shows that 56% (n = 328) women were eligible for a COVID-19 vaccine in pregnancy however remained unvaccinated, with no plans for vaccination.

**Fig. 3:**
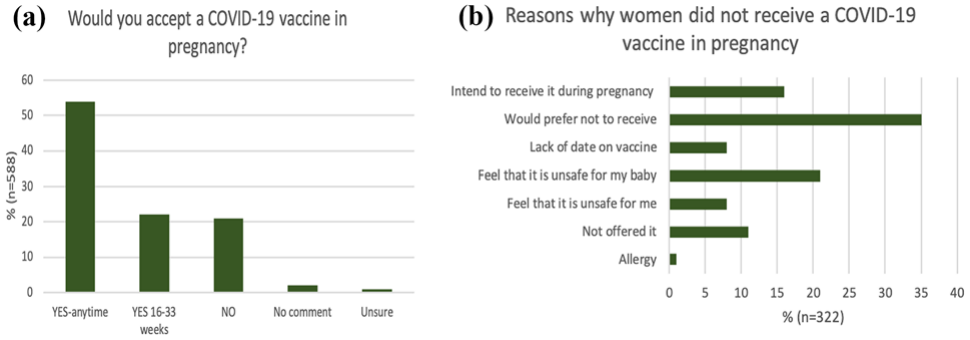
**a** Graphical representation of willingness to receive COVID-19 Vaccine in Pregnancy (n = 588), **b** If answered “No” to receiving a COVID-19 Vaccine in the last 4 months -women were asked why not? (n = 322 offered explanations)

Figure 3a shows the results of whether women would be happy to take a COVID-19 vaccine in pregnancy. It shows that 76% (n = 446) would be happy to receive a COVID-19 vaccine in pregnancy and 21% (n = 123) would not. Figure 3b shows reasons why women had not been vaccinated in the last four months (n = 322). Fifty women (16%) were planning on receiving a vaccine during pregnancy, 26 (8%) felt it was unsafe for them, 67 (21%) felt it was unsafe for their baby and 27 (8%) felt there was a lack of data on the vaccine.

## Discussion

Pregnancy is a risk factor for severe influenza and COVID-19 infection and pregnant women are more likely to be admitted to an ICU, require invasive ventilation and die than nonpregnant counterparts of a similar reproductive age [16, 17]. This study describes the uptake rates of influenza and COVID-19 vaccination and explores reasons for vaccine hesitancy in pregnant women in Ireland.

In the UK, seasonal influenza vaccine uptake in pregnant women has increased in the decade before the COVID-19 pandemic, but still remain low, with rates of 43.6% in 2020/21 taken from Public Health England [18]. This Irish based prospective cohort study found that the rate of influenza vaccine uptake in pregnancy has risen significantly to 57% (n = 377) from 39% in a similar study in the same hospital in 2016 [15]. One suspected major contributing factor to this rise in vaccine uptake is that the influenza vaccine is now provided free of charge to pregnant women in Ireland. Another possible reason for this improvement in influenza vaccination uptake is that women may be more accustomed to adult vaccination since the COVID-19 pandemic. A minority of women felt the influenza vaccine was unsafe in pregnancy (n = 12/588, 2%) and less still were unaware of the importance of vaccination in pregnancy (n = 6/588, 1%). This demonstrates that there is generally good public awareness of the recommended vaccines in pregnancy.

Most women reported receiving at least one COVID-19 vaccine (n = 488, 83%) since the pandemic began, with the highest proportion of women receiving one booster vaccine (n = 183, 31%). In December 2022, when the survey was conducted, women were eligible for a COVID-19 booster if it had been over four months since their last vaccine or COVID-19 infection and this timeframe features in our results section. Importantly, this advice has since changed with current Irish guidelines recommending to receive one COVID-19 booster vaccine in pregnancy, whereas two booster vaccines at six monthly intervals are reserved for those with a serious underlying risk factor/medical condition [8, 10]. However, vaccination rates during pregnancy remain low (22%, n = 132), despite 76% (n = 446) reporting that they would be happy accept a COVID-19 vaccine in pregnancy.

Our study illustrates that age, ethnic group, type of antenatal care received and the presence of co-morbidities can influence whether expectant mothers chose to have the influenza or COVID-19 vaccine. The demographic data markedly changes when comparing those who received one COVID-19 vaccine since the start of the pandemic versus those who received a COVID-19 vaccine in pregnancy. The youngest age group (age 15–19) were least likely to be vaccinated and vaccine uptake improved with increasing age. This is consistent with other studies which have shown that younger people are more likely to refuse or delay COVID-19 vaccination [19].

There was a 62% (n = 224/363) influenza vaccine uptake rate and a 25% (n = 89/363) COVID-19 vaccine uptake rate amongst the Irish population in pregnancy. Comparatively vaccine rates were lowest amongst the Irish Traveller community members, with 0% (n = 6) receiving either an influenza or COVID-19 vaccine in pregnancy. Although this group was underrepresented as only 6 members were surveyed, it is well known that compared to the general population, the Traveller community has substantial health inequalities and Travellers are at higher risk of vaccine-preventable diseases due to their social circumstances [20]. The highest vaccination rates during pregnancy were observed amongst those of Chinese ethnicity. Research has shown that China has one of the highest acceptance rates of COVID-19 vaccination in pregnancy, with an average of 77.4%, which may also account of our findings [19]. Other studies have shown that Black women were least likely to be vaccinated [21]. Our data showed average influenza vaccination rates amongst African women, however they had the lowest uptake of COVID-19 vaccine in pregnancy (5%, n = 1/19).

Important risk factors for severe influenza and COVID-19 infection include obesity, age, diabetes, hypertension, respiratory and cardiovascular diseases [22]. Our data shows an increase in influenza and COVID-19 vaccination uptake rates with increasing BMI and with the presence of co-morbidities. This may show that women at highest risk for severe disease are more amenable to vaccination. However, another possible explanation is that women with co-morbidities have a greater frequency of healthcare appointments and therefore may be more educated on the importance of vaccination.

Vaccine uptake was highest amongst those attending private and semi-private care when compared to public funded obstetric antenatal care. One possible explanation for this is that women have more individual time with their Consultant who can promote the benefits of vaccination and provide answers to safety concerns patients may have.

In our study, a small proportion of women said they did not receive an influenza or COVID-19 vaccine as they were not offered it. It is important that healthcare providers looking after pregnant women enhance vaccination rates by recommending eligible vaccines at each visit and focusing on the benefits to the health of the infant in conversations with patients [23]. Influenza vaccines in Ireland are usually offered between October and April. A recent UK study (ComFluCOV) has shown that concomitant vaccination with influenza and COVID-19 vaccines are equally effective at preventing disease when given together or alone [24]. Therefore, this strategy of dual vaccination may also be employed to reduce the burden on health-care services for vaccine delivery.

## Conclusion

Vaccination against viral illnesses is an important component of antenatal care. Our study shows that although Influenza vaccine uptake in pregnancy has improved in the last few years, COVID-19 vaccine uptake in pregnancy remains low. More targeted public health campaigns are required to promote vaccine safety and efficacy in pregnancy to improve vaccination uptake rates.
